# Cold-pressed minke whale oil reduces circulating LDL/VLDL-cholesterol, lipid oxidation and atherogenesis in apolipoprotein E-deficient mice fed a Western-type diet for 13 weeks

**DOI:** 10.1186/s12986-018-0269-8

**Published:** 2018-05-04

**Authors:** Mari Johannessen Walquist, Svein Kristian Stormo, Bjarne Østerud, Edel O. Elvevoll, Karl-Erik Eilertsen

**Affiliations:** 1 Norwegian College of Fishery Science, Faculty of Biosciences, Fisheries and Economics, UiT - The Arctic University of Norway, 9037 Tromsø, Norway; 2 Nofima, Muninbakken 9-13, pb 6122, 9291 Tromsø, Norway; 3 Faculty of Health Science, IMB, K.G Jebsen TREC, UiT - The Arctic University of Norway, 9037 Tromsø, Norway

**Keywords:** Atherosclerosis, Plaque, Lesions, *Balaenoptera acutorostrata*, Whale oil, Blubber, Gene expression, LDL-cholesterol, VLDL-cholesterol

## Abstract

**Background:**

Long-chain n3-polyunsaturated fatty acids (LC n3-PUFA) are well known for their anti-inflammatory activity and their impact on cardiovascular disease. Cold-pressed whale oil (CWO) has half the amount of LC n3-PUFA compared to cod liver oil (CLO). Still, there has been observed more pronounced beneficial effects on cardiovascular disease markers from intake of CWO compared to intake of CLO in human intervention studies. Extracts from CWO deprived of fatty acids have also been shown to display antioxidative and anti-inflammatory effects in vitro. The aim of this study was to investigate whether intake of a high-fat Western-type diet (WD) supplemented with CWO would prevent the development of atherosclerotic lesions in apolipoprotein E-deficient (ApoE^−/−^) mice.

**Methods:**

Seventy female ApoE^−/−^ mice were fed a WD containing 1% CWO, CLO or corn oil (CO). Atherosclerotic lesion formation, body and tissue weights, hepatic gene expression together with serum levels of LDL/VLDL-cholesterol, ox-LDL, total antioxidant status and various serum cardiovascular disease/proinflammatory markers were evaluated. Statistical analyses were performed using SPSS, and Shapiro-Wilk’s test was performed to determine the distribution of the variables. Statistical difference was assessed using One-Way ANOVA with Tukeys’ post hoc test or Kruskal-Wallis test. The hepatic relative gene expression was analysed with REST 2009 (V2.0.13).

**Results:**

Mice fed CWO had less atherosclerotic lesions in the aortic arch compared to mice fed CO. Levels of LDL/VLDL-cholesterol and ox-LDL-cholesterol were also markedly reduced whereas total antioxidant levels were enhanced in mice fed CWO compared to CO-fed mice. In addition, CWO-fed mice gained less weight and several hepatic genes involved in the cholesterol metabolism were up-regulated compared to CO-fed mice.

**Conclusion:**

In the present study mice fed a WD supplemented with 1% CWO had reduced formation of atherosclerotic lesions in the aortic arch, reduced serum LDL/VLDL-cholesterol and ox-LDL-cholesterol, increased serum total antioxidant status and reduced body weight compared to mice fed a WD supplemented with 1% CO.

**Electronic supplementary material:**

The online version of this article (10.1186/s12986-018-0269-8) contains supplementary material, which is available to authorized users.

## Background

Atherosclerosis is characterized by arterial lipid lesions in the intima of large arteries and the illness may be silent or symptomless for decades. Yet, atherosclerosis is the major underlying cause of several other cardiovascular diseases (CVD) such as unstable angina, myocardial infarction and stroke. Years of lipid accumulation in the vascular wall cause plaque formation and narrowing of the lumen. Plaque rupture may eventually lead to acute atherothrombosis preventing adequate blood flow to lung, heart and brain tissue [1, 2]. The link between atherosclerosis and inflammation has been comprehensively elucidated [3–7], and several cytokines and chemokines are key contributors in atherosclerotic progression [8–11]. The anti-inflammatory effects of long-chain n3-polyunsaturated fatty acids (LC n3-PUFA) are well known. Especially eicosapentaenoic acid (EPA) and docosahexaenoic acid (DHA) and how they contribute to mitigate atherosclerotic progression in coronary patients [12–17]. Oily fish is a good dietary source of EPA and DHA. Fish also contain other constituents such as proteins, amino acids, peptides and bioactive compounds that may contribute to the beneficial effects observed after fish intake [18–20].

The common minke whale (*Balaenoptera acutorostrata*) feeds on crustaceans and pelagic fish in the North Atlantic [21], and their thick layer of blubber is vital for thermal insulation, buoyancy and energy storage [22]. Blubber has been an important part of the diet to indigenous people in Arctic and Subarctic regions for centuries. Two decades ago, cold-pressed whale oil (CWO) and cod liver oil (CLO) were given as dietary supplements to healthy individuals as part of a larger study [23, 24]. The results indicated that the CWO group had beneficial effect on CVD markers and improved inflammatory effect, also when compared to the CLO group. The fatty acid (FA) composition of CWO and CLO differ in the amount of LC n3-PUFA. Whale blubber has 10.3% LC n3-PUFA, including 3.3% EPA, 1.7% docosapentaenoic acids (DPA) and 4.7% DHA while CLO has 25.1% LC n3-PUFA, including 9.5% EPA, 1% DPA and 13.5% DHA in CLO [24]. Recently, we demonstrated in an in vitro study with CWO deprived of fatty acids, that CWO contained extractable antioxidants and had anti-inflammatory activities associated with hitherto unidentified compound(s) [25].

In the present study, we investigated whether intake of a high-fat Western-type diet (WD) supplemented with CWO or refined whale oil (RWO) in combination with extracts from whale blubber would prevent the development of atherosclerotic lesions in Apolipoprotein E-deficient (ApoE^−/−^) mice.

## Methods

### Experimental animals and housing

Seventy-two pathogen-free female ApoE^−/−^-mice (B6.129P2-*Apoe*^*tm1 Unc*^ N11) were purchased from Taconic (Taconic M&B, Ry, Denmark). After arrival at the local animal facility all mice were earmarked and randomly allocated into 6 groups (*n* = 12) with equal numbers of cages per intervention (*n* = 4 cages/diet). Due to weight loss during the one-week acclimatization, two mice were excluded (*n* = 11 in the RWO-I and CWO groups). At the start of the experiment, the mice were 6 weeks of age with a body weight range of 16 to 21 g. Mice provided with water and pelleted feed ad libitum for 13 weeks were kept in ventilated cages placed in the same room in a conventional laboratory animal unit. The temperature and relative humidity were 21 °C and 55% on a 12-h day/night cycle (light: 0600 to 1800 h). The cages and bedding were changed once a week. At the end of the study all mice were feed-deprived for 3 h prior to euthanizing by carbon dioxide inhalation. Blood was drawn by cardiac puncture and serum was prepared and frozen at − 80 °C. Cardiac, hepatic, renal, splenic and adipose tissues were dissected out, weighed, snap frozen and stored at − 80 °C.

### Preparation of dietary oils

Freshly frozen blubber from common minke whale was provided by Ellingsen Seafood AS (Skrova, Norway). The blubber was ground once before centrifugation at < 2000×g (< 40 °C). After centrifugation the oily top-layer, hereafter referred to as CWO in this study, was collected and 250 g CWO was extracted in 800 ml methanol/dichloromethane (1:1). After phase separation, most of the lipids (oil) was in the fraction containing dichloromethane. The better part of the dichloromethane was removed by a rotary evaporator and the remaining solvent was removed during nitrogen flushing for 48 h. After extraction of the CWO, the oil that was left is referred to as RWO. For the methanol phase, containing more polar compounds extracted from CWO, residual dichloromethane and oil were removed with a rotary evaporator followed by 3 × 200 ml heptane liquid-liquid extraction. Subsequently, the methanol in this fraction was evaporated to almost dryness and was finally removed by flushing the sample with nitrogen for 48 h (extract I). The remnant of whale blubber after the initial removal of oil top layer was next extracted according to the same protocol as for CWO. Due to the low level of oil in the cold pressed blubber, refined whale oil was not collected from this dichloromethane fraction. Extract II (whale blubber extract) was also flushed with nitrogen for 48 h to remove all traces of solvents. Oils and extracts were flushed with nitrogen and stored at − 20 °C prior to further analyses. Commercial CLO was bought from Orkla Health [26] whereas CO was bulk oil provided by the diet manufacturer.

### Experimental diets

The mice were fed six different high-fat WD (modified from EF D-12079, *ssniff Spezialdiäten GmbH*). The six diets were supplemented with 1% of six different PUFA-rich oils as indicated in Table 1. Diet A was a control diet containing 1% CO, diet B contained 1% commercial CLO [26], diet C contained 1% RWO, diet D contained 1% RWO-I, diet E contained 1% RWO-II, and diet F contained 1% CWO. Apart from the PUFA-source the diets were identical, however to compensate for the higher cholesterol content of CLO compared to the other dietary oils used in this study, a smaller amount of cholesterol was added to the CLO diet (1.47 g/kg vs 1.5 g/kg for the rest of the diets, Table 1). The experimental diets were stored at − 20 °C and the feed was changed every week.

**Table 1:** Content of diets g/kg

	CO Diet A	CLO Diet B	RWO Diet C	RWO-I Diet D	RWO-II Diet E	CWO Diet F
Corn oil	10					
Cod liver oil		10				
Refined whale oil			10			
Refined whale oil + extract I				10		
Refined whale oil + extract II					10	
Cold-pressed whale oil						10
Casein	195	195	195	195	195	195
Corn starch	50	50	50	50	50	50
Maltodextrin. 10 DE	99.4	99.4	99.4	99.4	99.4	99.4
Sucrose	340	340	340	340	340	340
Celullose powder	50	50	50	50	50	50
DL-Methionine	3	3	3	3	3	3
AIN mineral premix	35	35	35	35	35	35
Vitamin premix	10	10	10	10	10	10
Calcium carbonate	4	4	4	4	4	4
Choline Cl	2	2	2	2	2	2
Butylated hydroxytoluene	0.1	0.1	0.1	0.1	0.1	0.1
Cholesterol	1.5	1.47	1.5	1.5	1.5	1.5
Butter fat	200	200	200	200	200	200

### Analysis of atherosclerotic plaque

Immediately after blood drainage, all mice were perfused through the left ventricle with sterile saline (0.9%), until no residual blood was apparent in the perfusate (approximate 5 min perfusion). The entire aorta (proximal ascending the aorta to bifurcation of the iliac arteries) was cleaned in situ of periadventitial fat, dissected and fixed in 1% paraformaldehyde solution. Finally, the aorta was stained by Oil Red O staining, opened longitudinally and *en face-*mounted on slides as previously described [27]. After 48 h rest, the slides were scanned with a high-resolution scanner. The lesion areas were evaluated using ImageJ software [28] and the extent of atherosclerosis was reported as the percentage of the total area of a given aortaor an aortic region occupied by atherosclerotic lesions.

### Total RNA extraction

After perfusion of the mice, livers were removed, weighed and frozen in liquid nitrogen before storage at − 80 °C until extraction. One hundred mg liver tissue was homogenized in 1 ml Trizol (Life Technologies) by bead milling (Precellys 24, Bertin Technologies). The samples were incubated on ice for 30 min followed by 20 min centrifugation at 12000×g at 4 °C. The samples were precipitated overnight with isopropanol and centrifuged for 21,000×g for 30 min at 4 °C. Pellets were dissolved in RNA storage solutions and RNA was stored at − 80 °C until further processing. Total RNA concentration was measured using Qubit fluorometer 1.0 (Life Technologies) and the quality tested with Agilent 2100 Bioanalyzer (Agilent Technologies, Inc). The RNA Integrity Factor was of 7.6–9.3 for the samples used.

### Reverse transcription and quantitative real-time PCR

High capacity cDNA Reverse Transcription kit (4,368,813, Applied Biosystems) was used to make 3 triplicates of reverse transcriptase (50 ng total RNA in 20 μl). Quantitative RT-PCR was used to analyse 4 μl cDNA per 20 μl reaction using TaqMan® Fast Universal PCR Master mix (4,352,042, Applied Biosystems) and with predesigned TaqMan® Gene Expression assays (Additional file 1: Table S1). The 96-wells plates were run at ABI Prism 7500 Fast cycler (Applied Biosystems) using the pre-set amplification profile (95 °C 20s, 40 × 95 °C 3 s and 60 °C 30s). The most stably expressed endogenous reference genes, Hypoxanthine-guanine phosphoribosyltransferase 1 *(Hprt1)* and TATA-Box Binding Protein (*Tbp)*, were selected using TaqMan Array Mouse Endogenous Control Assay (4,426,701, Applied Biosystems). The geometric mean of these reference genes was used to normalize gene expressions. Inter plate calibrator and none-template controls were included in all assays.

### Serum analyses

Serum cholesterol, low-density lipoprotein cholesterol and very low-density lipoprotein (LDL/VLDL), glucose, triacylglycerol (TAG), total protein concentrations, uric acid, non-esterified fatty acids (NEFA; D07940, Dialab, Austria) and total antioxidant status (TAS; NX2332 Randox Total Antioxidant Status, Randox Laboratories Ltd., UK) were analysed using conventional enzymatic kits and a MaxMat PLII bioanalyser (MaxMat PL, Montpellier, France). Unless otherwise stated all kits were from MaxMat PL (Montpellier, France). Serum oxidized LDL (Ox-LDL) was quantified in duplicates according to the manufacturers’ instruction with a murine ELISA-kit (E90527Mu, USCN Life Science Inc., Texas, US). Serum samples were analysed in duplicates for the following cytokines: interferon gamma (IFNγ), interleukin 10 (IL-10), interleukin 1 beta (IL-1β), interleukin 2 (IL-2), interleukin 5 (IL-5), interleukin 6 (IL-6), keratinocyte chemoattractant growth-regulated oncogene (KC-GRO), and tumour necrosis factor alpha (TNF-α). These analyses were performed according to the manufacturers’ instructions using a MSD Mouse Proinflammatory panel 1 V-Plex kit (MULTI-ARRAY®, Meso Scale Discovery, Gaithersburg, MD).

### Statistical analyses

All of the statistical analyses were performed using IBM SPSS Statistics for Macintosh (Release 22.0.0.0, SPSS, Inc., Chicago, IL, US). The Shapiro-Wilk’s test was performed to determine the distribution of the variables and non-normally distributed variables were log-transformed before statistical analysis. Statistical difference was assessed using One-Way ANOVA with Tukeys’ post hoc test or Kruskal-Wallis test. The relative gene expression was analysed with REST 2009 (V2.0.13) [29]. A value of *p* < 0.05 was considered statistically significant.

## Results

**Fig. 1:**
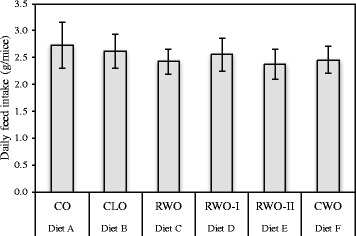
Average daily feed intake (g/mice) of female apolipoprotein E-deficient mice fed high-fat diets supplemented with different oils for 13 weeks. The results are presented as mean ± SD. CO (*n* = 11), CLO (*n* = 12), RWO (*n* = 12), RWO-I (*n* = 11), RWO-II (*n* = 12), CWO (*n* = 11)

**Fig. 2:**
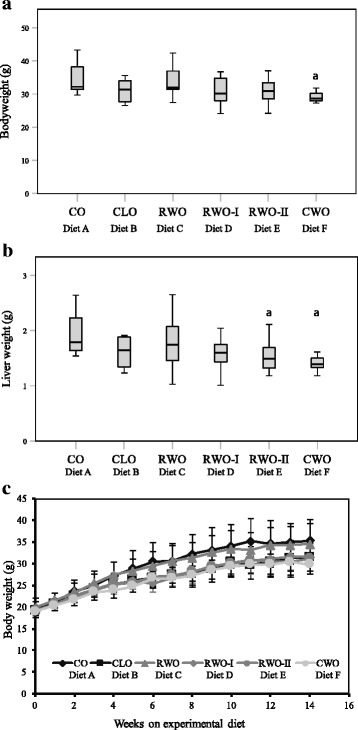
Final body weight (**a**), liver weight (**b**) and growth curves (**c**) of female apolipoprotein E-deficient mice fed high-fat diets supplemented with difference oils for 13 weeks. Data are presented as box plots representing a 95% confidence interval (CI) for the median (**a** and **b**) and as bars representing mean ± SD (**c**). **a** denotes significant different from CO (diet A), *p* < 0.05. CO (*n* = 11), CLO (*n* = 9), RWO (*n* = 11), RWO-I (*n* = 11), RWO-II (*n* = 12), CWO (*n* = 10)

The general physical health and weight gain appeared normal for all mice except one CO-fed mouse (euthanized due to weight loss) during the 13 weeks with experimental diets. The average daily feed intake (g/mice) was equal for all of the 6 diet groups (Fig. 1). Still, the mice fed CWO gained less body weight compared to the mice fed CO (Fig. 2a). Liver weights in mice fed CWO and RWO-II were lower than the liver weights in mice fed CO (Fig. 2b). These significant differences were also present for the relative liver weights. The amount of white adipose tissue was also lower in the mice fed CWO compared to the mice fed CO (1.44 g ± 0.12 vs 2.24 ± 0.2 respectively), however, not significant (*p* = 0.078). There were no apparent differences in tissue sizes between the groups for heart, kidney or spleen (Table 2). The growth curves for all of the experimental diets are presented in Fig. 2c.

**Table 2:** Serum and organ characteristics in female apolipoprotein E-deficient mice fed high-fat Western-type diets supplemented with different oils for 13 weeks

	CO Diet A	CLO Diet B	RWO Diet C	RWO-I Diet D	RWO-II Diet E	CWO Diet F
TAG (mmol/l)	2.18 ± 0.52	2.36 ± 0.61	2.35 ± 0.68	2.04 ± 0.49	2.03 ± 0.75	2.04 ± 0.74
Glucose (mmol/l)	14.5 ± 2.40	12.4 ± 1.84	15.2 ± 3.25	13.2 ± 3.87	13.6 ± 2.56	13.8 ± 2.22
Uric acid (mmol/l)	409 ± 165	399 ± 168	541 ± 211	505 ± 250	414 ± 219	354.4 ± 132
Proteins (g/l)	83.1 ± 18.2	73.3 ± 13.5	87.7 ± 16.4	85.1 ± 21.6	81.9 ± 16.7	79.3 ± 11.8
NEFA (mmol/l)	1.44 ± 0.24	1.66 ± 0.19	1.64 ± 0.25	1.56 ± 0.29	1.56 ± 0.34	1.78 ± 0.60
Spleen (g)	0.17 ± 0.05	0.17 ± 0.02	0.21 ± 0.07	0.20 ± 0.07	0.19 ± 0.05	0.17 ± 0.03
Kidney (g)	0.16 ± 0.03	0.15 ± 0.01	0.16 ± 0.02	0.16 ± 0.03	0.15 ± 0.02	0.15 ± 0.03
Heart (g)	0.15 ± 0.02	0.13 ± 0.01	0.15 ± 0.02	0.14 ± 0.02	0.14 ± 0.02	0.13 ± 0.01
Adipose (g)	2.24 ± 0.67	1.60 ± 0.58	2.03 ± 0.74	1.71 ± 0.67	2.08 ± 0.80	1.44 ± 0.38
IFNγ (pg/ml)	0.79 ± 0.97	1.26 ± 1.90	0.43 ± 0.14	0.57 ± 0.42	0.60 ± 0.51	0.45 ± 0.19
IL-10 (pg/ml)	40.7 ± 6.64	40.4 ± 17.1	40.4 ± 12.6	62.8 ± 88.6	39.1 ± 9.66	39.4 ± 10.5
IL-1β (pg/ml)	2.81 ± 1.49	2.61 ± 0.92	1.41 ± 2.68	3.29 ± 1.92	2.57 ± 2.09	3.20 ± 1.36
IL-2 (pg/ml)	1.03 ± 0.33	0.94 ± 0.40	1.13 ± 0.40	1.09 ± 0.61	1.15 ± 0.44	1.17 ± 0.27
IL-5 (pg/ml)	10.9 ± 5.54	7.82 ± 3.60	9.20 ± 4.83	9.28 ± 4.97	7.33 ± 3.19	10.2 ± 3.93
IL-6 (pg/ml)	61.1 ± 64.3	43.4 ± 28.1	50.6 ± 24.4	73.1 ± 55.5	58.4 ± 52.1	46.5 ± 28.5
KC/GRO (pg/ml)	195 ± 52.9	172 ± 43.5	168 ± 52.3	173 ± 81.2	146 ± 32.7	156 ± 63.1
TNFα (pg/ml)	25.3 ± 13.4	21.6 ± 5.90	20.1 ± 5.66	21.6 ± 10.3	24.6 ± 7.11	19.11 ± 4.80

Data are presented as mean ± SD. CO (*n* = 11), CLO (*n* = 9), RWO (*n* = 11), RWO-I (*n* = 11), RWO-II (*n* = 12), CWO (*n* = 10). None of the values for any group in this table are significantly different from values in any other group

### Atherosclerotic lesions

**Fig. 3:**
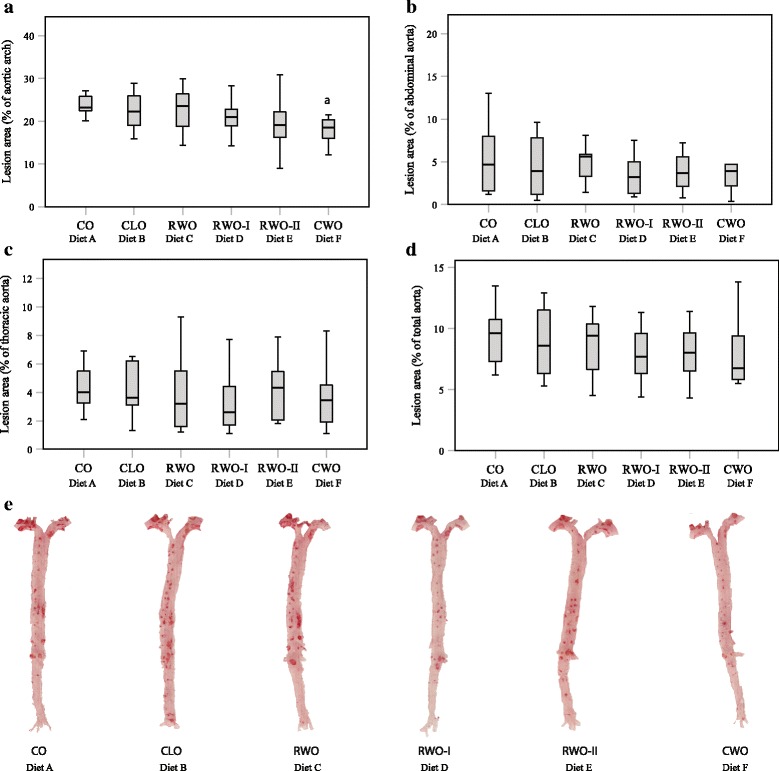
Box plot of atherosclerotic plaque burden expressed as aortic area percentage covered by lipid Oil Red O staining in female apolipoprotein E-deficient mice fed high-fat diets supplemented with different oils for 13 weeks (**a**-**d**). Data are presented as a 95% confidence interval (CI) for the median, and **a** denotes significant difference from mice fed CO (diet A) with *p* < 0.05. CO (*n* = 11), CLO (*n* = 9), RWO (*n* = 11), RWO-I (*n* = 11), RWO-II (*n* = 12), CWO (*n* = 10). (**e**) shows a representative aorta from each group stained with Oil Red O Staining

Five mice were removed from the dataset for atherosclerotic lesion analyses due to methodological artefacts or severe calcification of the abdominal aorta (from the renal arteries down to the iliac bifurcation) despite minimal lesion formation in the aortic arch region. The omitted mice were; three mice fed CLO (*n* = 9), one mouse fed RWO (*n* = 11) and one mouse fed CWO (*n* = 10). The lesion area in the aortic arch was lower in mice fed CWO compared to the lesion area of the aortic arch in mice fed CO (Fig. 3a). No significant between-group differences were observed in other regions in the aorta (abdominal part, thoracic part or even for the total aorta) (Fig. 3b-d respectively).

### Serum antioxidant status, serum LDL/VLDL-cholesterol levels and serum ox-LDL levels

**Fig. 4:**
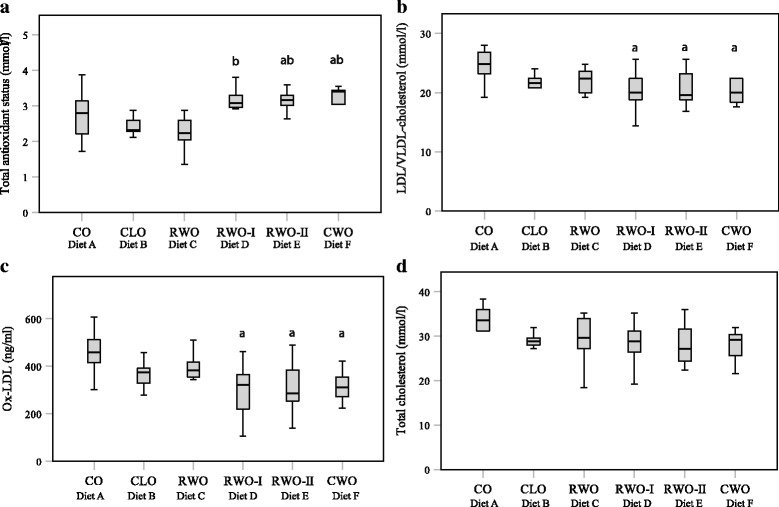
Box plot of serum total antioxidant status (**a**), LDL/VLDL-cholesterol levels (**b**), ox-LDL-cholesterol levels (**c**) and total cholesterol (**d**) in female apolipoprotein E-deficient mice fed high-fat diets supplemented with different oils for 13 weeks. Data are presented with 95% confidence interval (CI), and **a** denotes significant different from mice fed CO (diet A) whereas **b** denotes significant difference from mice fed CLO (diet B) with p < 0.05. CO (*n* = 11), CLO (*n* = 9), RWO (*n* = 11), RWO-I (*n* = 11), RWO-II (*n* = 12), CWO (*n* = 10)

Serum TAS was higher in mice fed CWO and RWO-II compared to mice fed CO (Fig. 4a). TAS was also higher in mice fed RWO-I, RWO-II and CWO when compared to TAS in mice fed CLO (Fig. 4a). Serum LDL/VLDL-cholesterol levels and serum ox-LDL-cholesterol levels were lower in mice fed RWO-I, RWO-II and CWO compared to these levels in mice fed CO (Fig. 4b and c). Total cholesterol (Fig. 4d) and serum triglycerides did not differ between mice fed CO and the other diet groups; neither did serum glucose or cytokines levels (Table 2).

### Gene expression

Hepatic gene expression assessments included genes involved in cholesterol and lipid metabolism, antioxidant defence and in the inflammatory response (Table 3a and b). In mice fed CLO (diet B), expression of the genes *ATP binding cassette, sub-family G member 8* (*Abcg8*) and *Scavenger receptor class B member 1* (*Sr-b1*) was enhanced (1.32 95% CI [0.64–2.61] and 1.29 95% CI [0.76–2.29]) whereas *Very low density lipoprotein receptor* (*Vldlr*) gene expression was reduced (0.75 95% CI [0.34–1.59]) compared to mice fed CO (diet A). In mice fed RWO (diet C), *Peroxisome proliferator-activated receptor-α* (*Pparα*) gene expression was enhanced (1.41 95% CI [0.67–2.66]) compared to mice fed CO. In mice fed RWO-I expression of the genes *Abcg8, Sr-b1* and *Pparα* was increased (1.43 95% CI [0.67–2.05], 1.43 95% CI [0.78–2.46] and 1.79 [0.77–3.81] respectively) compared to mice fed CO. The most dramatic changes in gene expression, when compared to mice fed CO, were observed in mice fed RWO-II and where gene expression of *Abcg8* (1.40 95% CI [0.65–2.66]), *ATP binding cassette, sub-family G member 5* (*Abcg5*) (1.28 95% CI [0.51–2.22]), *Cytochrome P450 7A1* (*Cyp7a1*) (1.95 95% CI 1.95^*^ [0.35–8.92]), 3*-hydroxy-3-methyl-glutaryl-Coenzyme A reductase* (*Hmgcr*) (1.31 [0.62–2.67]), *Sr-b1* (1.26 95% CI [0.58–2.25]), *Pparα* (1.39 95% CI [0.74–2.77]) and Peroxisome proliferator-activated receptor*-*γ (*Pparγ*) (1.26 95% CI [0.70–2.01]) was increased. In mice fed CWO, gene expression of *Abcg5* (1.46 95% CI [0.59–2.58]), *Sr-b1* (1.29 95% CI [0.66–2.27]) and *Pparα* (1.39 95% CI [0.60–3.09]) was enhanced compared to mice fed CO. Mice from the same group also had elevated gene expression of *Abcg8* (1.31 95% CI [0.62–2.83]), however, not significantly (*p* = 0.064). Gene expression of *Pparγ* was increased (1.25 95% CI [0.80–1.83]) in mice fed RWO compared to mice fed CLO. In mice fed RWO-I the gene expression of *Ppar*α (1.57 95% CI [0.77–3.30]) was increased whereas gene expression of *Fatty acid synthase* (*Fasn*) (0.59 95% CI [0.19–2.66]) was reduced compared to mice fed CLO. Further, when comparing to mice fed CLO, it was observed that mice fed RWO-II had increased gene expression of *Cyp7a1* (2.23 95% CI 0.35–17.1]), *Vldlr* (1.52 95% CI [0.76–2.97]) and *Pparγ* (1.37 95% CI [0.87–2.06]). Finally, in mice fed CWO gene expression of *IL-6* was reduced (0.51 95% CI [0.07–3.67]) while gene expression of *Uncoupling protein 2* (*Upc2*) (1.41 95% CI [0.69–2.94]) was increased compared to mice fed CLO. Expression of all other genes were unaffected by the dietary interventions (Table 3a and b).

**Table 3:** Hepatic gene expression in female apolipoprotein E-deficient mice fed high-fat Western-type diets supplemented with different oils for 13 weeks

a)	CLO Diet B [95% CI]	RWO Diet C [95% CI]	RWO-I Diet D [95% CI]	RWO-II Diet E [95% CI]	CWO Diet F [95% CI]
*Tnfα*	1.03 [0.24–2.59]	0.76 [0.16–2.35]	0.79 [0.11–5.21]	0.76 [0.14–2.38]	1.18 [0.16–13.9]
*Mcp1*	1.01 [0.37–2.70]	0.94 [0.35–2.59]	0.79 [0.11–3.15]	0.80 [0.26–2.78]	0.92 [0.23–3.27]
*Il-6*	1.39 [0.42–5.14]	0.87 [0.26–2.52]	1.09 [0.26–3.07]	1.18 [0.29–4.20]	0.79 [0.11–4.22]
*Icam1*	1.11 [0.70–1.704]	1.08 [0.65–0.21]	1.09 [0.462–2.46]	0.74 [0.02–1.87]	1.18 [0.52–2.25]
*Vcam1*	0.85 [0.47–1.38]	0.86 [0.55–1.31]	0.88 [0.57–1.40]	0.89 [0.50–1.29]	0.85^*^ [0.50–1.39]
*Pon2*	0.96 [0.76–1.22]	0.99 [0.76–1.29]	0.98 [0.83–1.33]	0.96 [0.81–1.12]	1.00 [0.73–1.25]
*Nfe212*	0.98 [0.62–1.47]	1.01 [0.64–2.04]	0.93 [0.57–1.52]	1.01 [0.65–1.48]	1.06 [0.61–1.76]
*Upc2*	0.96 [0.43–1.50]	1.03 [0.49–1.58]	1.03 [0.36–2.33]	1.11 [0.45–2.01]	1.35 [0.49–3.02]
*Abcg5*	1.15 [0.45–1.90]	1.06 [0.40–1.71]	1.08 [0.31–2.42]	1.28^*^ [0.51–2.22]	1.46^*^ [0.59–2.58]
*Abcg8*	1.32^*^ [0.64–2.61]	1.12 [0.56–2.16]	1.43^*^ [0.67–2.05]	1.40^*^ [0.65–2.66]	1.31 [0.62–2.83]
*Acat2*	1.00 [0.64–1-66]	1.02 [0.50–2.22]	1.02 [0.57–1.83]	1.09 [0.48–2.30]	0.91 [0.50–1.52]
*Cyp7a1*	0.88 [0.11–4.85]	1.19 [0.34–4.47]	1.48 [0.23–7.65]	1.95^*^ [0.35–8.92]	1.19 [0.37–6.34]
*Hmgcr*	1.16 [0.62–2.12]	1.11 [0.60–2.22]	1.12 [0.55–2.38]	1.31^*^ [0.62–2.67]	1.04 [0.49–2.40]
*Ldlr*	1.13 [0.57–2.55]	1.14 [0.61–2.09]	1.27 [0.57–2.67]	1.19 [0.61–2.38]	1.03 [0.40–2.55]
*Sr-b1*	1.29^*^ [0.76–2.29]	1.23 [0.67–2.30]	1.43^*^ [0.78–2.46]	1.26^*^ [0.58–2.25]	1.29^*^ [0.66–2.27]
*Vldlr*	0.75^*^ [0.34–1.59]	0.92 [0.46–1.75]	0.87 [0.23–2.05]	1.14 [0.58–2.11]	0.79 [0.30–1.61]
*Pparα*	1.14 [0.53–2.22]	1.41^*^ [0.67–2.66]	1.79^*^ [0.77–3.81]	1.39^*^ [0.74–2.77]	1.39^*^ [0.60–3.09]
*Pparγ*	0.92 [0.54–1.39]	1.15 [0.67–1.71]	1.16 [0.52–2.21]	1.26^*^ [0.70–2.01]	1.12 [0.47–9.86]
*Fasn*	1.21 [0.35–5.39]	1.01 [0.20–4.31]	0.72 [0.22–3.60]	0.94 [0.21–4.88]	0.81 [0.20–3.74]
b)	CO Diet A [95% CI]	RWO Diet C [95% CI]	RWO-I Diet D [95% CI]	RWO-II Diet E [95% CI]	CWO Diet F [95% CI]
*Tnfα*	0.97 [0.39–4.10]	0.74 [0.36–1.69]	0.77 [0.15–4.35]	0.74 [0.29–1.69]	1.15 [0.25–11.0]
*Mcp1*	0.99 [0.37–2.70]	0.93 [0.44–2.15]	0.78 [0.09–3.45]	0.79 [0.27–2.29]	0.92 [0.22–2.73]
*Il-6*	0.72 [0.20–2.43]	0.63 [0.15–2.25]	0.79 [0.16–2.62]	0.85 [0.16–3.70]	0.51^*^ [0.07–3.67]
*Icam1*	0.90 [0.59–1.44]	0.98 [0.69–1.90]	0.98 [0.42–2.25]	0.67 [0.02–1.59]	1.06 [0.45–1.89]
*Vcam1*	1.18 [0.72–2.14]	1.01 [0.57–2.01]	1.03 [0.57–1.98]	1.05 [0.50–1.98]	1.00 [0.51–2.09]
*Pon2*	1.05 [0.82–1.32]	1.04 [0.77–1.39]	1.02 [0.79–1.39]	1.00 [0.78–1.26]	1.05 [0.77–1.39]
*Nfe212*	1.03 [0.68–1.62]	1.04 [0.74–2.14]	0.95 [0.55–1.45]	1.03 [0.73–1.40]	1.09 [0.70–1.75]
*Upc2*	1.04 [0.67–2.35]	1.07 [0.71–1.54]	1.07 [0.59–2.14]	1.15 [0.63–1.98]	1.41^*^ [0.69–2.94]
*Abcg5*	0.87 [0.53–2.21]	0.93 [0.57–1.47]	0.94 [0.24–2.07]	1.12 [0.64–1.89]	1.27 [0.63–2.22]
*Abcg8*	0.76^*^ [0.38–1.58]	0.85 [0.46–1.71]	1.09 [0.58–2.14]	1.06 [0.53–2.06]	0.99 [0.51–2.14]
*Acat2*	0.99 [0.60–1.55]	1.01 [0.48–2.13]	1.01 [0.56–1.94]	1.09 [0.45–2.46]	0.91 [0.49–1.55]
*Cyp7a1*	1.14 [0.21–8.90]	1.36 [0.35–9.16]	1.69 [0.23–14.4]	2.23^*^ [0.35–17.1]	1.36 [0.37–10.5]
*Hmgcr*	0.86 [0.47–1.62]	0.96 [0.62–1.63]	0.97 [0.58–1.72]	1.13 [0.60–2.18]	0.90 [0.50–2.00]
*Ldlr*	0.89 [0.39–1.76]	1.01 [0.49–1.78]	1.12 [0.30–2.77]	1.05 [0.49–1.98]	0.91 [0.36–2.09]
*Sr-b1*	0.77^*^ [0.44–1.32]	0.95 [0.54–1.61]	1.10 [0.65–1.74]	0.97 [0.49–1.57]	0.99 [0.54–1.59]
*Vldlr*	1.34^*^ [0.63–2.99]	1.23 [0.62–2.37]	1.17 [0.30–2.77]	1.52^*^ [0.76–2.97]	1.05 [0.39–2.46]
*Pparα*	0.88 [0.45–1.89]	1.23 [0.53–2.24]	1.57^*^ [0.77–3.30]	1.21 [0.76–2.97]	1.22 [0.61–2.91]
*Pparγ*	1.09 [0.72–1.84]	1.25^*^ [0.80–1.83]	1.26 [0.56–2.27]	1.37^*^ [0.87–2.06]	1.22 [0.59–10.6]
*Fasn*	0.83 [0.19–2.85]	0.84 [0.14–2.99]	0.59^*^ [0.19–2.66]	0.78 [0.18–3.34]	0.66 [0.15–1.27]

Relative comparison of mice fed CO to the other diets group (**a**), and relative comparison of mice fed CLO to the other diets group (**b**). 95% confidence interval [95% CI]. The relative gene expression was analysed with REST 2009 and ^*^denotes significant difference (*p* < 0.05). CO (*n* = 11), CLO (*n* = 9), RWO (*n* = 11), RWO-I (*n* = 11), RWO-II (*n* = 12), CWO (*n* = 10)

## Discussion

The main purpose of this study was to investigate whether dietary consumption of WD supplemented with CWO (diet F) would prevent atherosclerotic development in ApoE^−/−^-mice. Recently, our group demonstrated that extracts from CWO had antioxidant and anti-inflammatory activities not related to the content of LC n3-PUFA present [25]. When CWO was given as a dietary supplement to healthy volunteers, beneficial effects on CVD markers and improved anti-inflammatory effect were observed [23, 24]. Herein, WD was supplemented with 1% of CWO, or 1% RWO enriched with two different extracts from whale blubber using CO (diet A) and CLO (diet B) as control diets based on previous results [23–25].

This study indicates that a physiologically obtainable dietary supplementation with CWO prevents WD-induced atherogenesis. Mice fed a diet supplemented with 1% CWO for 13 weeks had significantly lower development of atherosclerotic lesions in the aortic arch compared to mice fed WD supplemented with 1% CO. There was, however, no differences in lesion formation in less lesion-prone parts of the aorta, such as the thoracic aorta, abdominal aorta or the total aorta. It was also evident that serum levels of LDL/VLDL-cholesterol and ox-LDL-cholesterol were markedly reduced, whereas serum TAS was significantly enhanced in CWO-fed mice compared to mice fed CO or CLO. This is in accordance with our recent demonstration that extracts from whale blubber have in vitro antioxidative effects [25]. A similar anti-atherosclerotic effect has previously been reported in ApoE^−/−^-mice fed seal oil [27]. It is, however, worthy to note that the seal oil was given in combination with extra virgin olive oil known to contain protective antioxidants. Antioxidants prevent lipid peroxidation and oxidative damage [30]. Indeed, the results herein suggest that CWO does not need a further addition of antioxidants, something which was shown by the high antioxidative capacity observed in our in vitro study [25]. Together, the increased TAS levels and lower ox-LDL-cholesterol levels explain the observed reduction of atherosclerotic lesions in the aortic arch observed in the CWO-fed mice. In addition, the final body weights were lower in the CWO-fed mice even though the feed intake was equal. At the same time, no differences in serum TAS levels or final body weights were observed between the CLO- and the CO-fed mice.

Although cholesterol is crucial for all mammalian cells, it is well known that increased circulating levels of LDL-cholesterol is a major risk factor for atherosclerotic CVD. In recent years much effort has been put into reducing circulation cholesterol levels in high-risk patients [31]. The liver is the major site for cholesterol synthesis, and de novo homeostasis is regulated by intestinal absorption and faecal and biliary excretion [32]. To shed light on the mechanisms involved in the observed LDL/VLDL-lowering effect of CWO, several receptors and enzymes involved in the cholesterol metabolism were investigated by means of gene expression analysis. In our study, expression of the hepatic genes *Abcg5*, *Abcg8* (*p* = 0.064), *Sr-b1* and *Pparα* were moderately up-regulated in mice fed CWO compared to mice fed CO. All these genes encode for proteins that are important for cholesterol homeostasis. The liver receptor SR-B1 is involved in the selective uptake of cholesteryl esters (CE) from high density lipoproteins (HDL) both in humans and mice [33]. The hepatic expression of *Sr-b1* has been shown to play an atheroprotective role associated with its impact on circulation cholesterol levels [34]. CE is further converted into bile by the obligate heterodimer ABCG5 and ABCG8 located in the hepatocytes [35]. Overexpression of *Abcg5*/*Abcg8* has been shown to increase biliary cholesterol excretion, together with reduced levels of liver inflammatory markers [36]. This effect was lower in mice fed CLO as only expression of *Abcg8* and *Sr-b1* were increased compared to the CO-control mice. This corresponds well with the increased expression of *Abcg5* together with reduced circulating levels of both LDL/VLDL-cholesterol and ox-LDL-cholesterol observed in CWO-fed mice. At the same time, these mice displayed no changes in inflammatory markers as assessed by hepatic gene expression and serum protein levels. PPARα is a nuclear receptor involved in transport, uptake, oxidation and reduction of FA and the triglyceride synthesis [37], and may also be involved in the observed effects. Natural ligands for PPARα include n3-PUFA and it is possible that the diets containing the marine oils may activate PPARα and contribute to the beneficial effects observed in this study.

We were further interested in whether the beneficial effects from CWO might be obtained via reconstitution of the extract(s) with RWO. Hence, two different extracts were prepared and reconstituted with RWO and the mice received three addition diets supplemented with (i) RWO (diet C), (ii) RWO + extract I (RWO-I, diet D) and (iii) RWO + extract II (RWO-II, diet E). This would indicate whether the anti-inflammatory activity was associated with the fatty acids (RWO), the water-soluble components in the whale oil (RWO-I) or the water-soluble components in the residual whale blubber (RWO-II). Indeed, compared to corn oil both RWO-I and RWO-II also reduced serum LDL/VLDL-cholesterol and ox-LDL-cholesterol concentration, whereas serum total antioxidant status was increased. Mice fed RWO-I had increased hepatic expression of *Abcg8, Sr-b1* and *Pparα*, and mice fed RWO-II had even more affected genes as the hepatic expression of *Abcg5*, *Abcg8*, *Cyp7al*, *Hmgcr*, *Sr-b1*, *Pparα* and *Pparγ*, were all increased. The mice fed RWO-II also had increased expression of *Cyp7al*, *Vldlr* and *Pparγ* compared to the CLO-fed mice. However, no changes were observed for atherosclerosis (not significantly reduced) or inflammation markers. This indicated that the extracts affected the same metabolic processes, however, the phenotypic effects were moderate compared to the effects observed in CWO-fed mice.

Due to the lower content of EPA and DHA in whale blubber compared to fish oils, we expected, and observed, unaltered serum TAG levels. Unaltered serum TAG levels have been observed in several other studies where ApoE^−/−^-mice were given comparable amounts of EPA, DPA and DHA [38–40]. Efficient reduction of serum TAG levels requires EPA, DPA and DHA supplementation in pharmaceutical doses which may be difficult to obtain through diet [41]. Whale blubber oil is known to contain large amounts of DPA compared to fish oil, and some studies have indicated that DPA may be the most important LC n3-PUFA when it comes to protection of CVD [42, 43]. Taken together these findings may imply that dietary CWO promotes cholesterol clearance from the circulation, increased clearance of cholesterol in the liver elevated TAS levels, lowered oxidation of LDL-cholesterol, all conducting to mitigate atherosclerosis.

## Conclusion

In the present study, we observed that mice fed WD with 1% CWO had reduced formation of atherosclerotic lesions in the aortic arch compared to mice fed WD with 1% CO. In addition, CWO-fed mice had reduced serum LDL/VLDL-cholesterol, ox-LDL-cholesterol and body weight and increased serum total antioxidant status compared to CO-fed mice. Our study adds novel insight into the putative protective mechanisms of dietary CWO supplementation in CVD, including activation of endogenous antioxidant responses, inhibition of LDL oxidation, attenuation of the diet-induced hypercholesterolemia and reduced aortic atherogenesis. These results may inform future dietary recommendations to reduce CVD and promote public health.

## Additional file

Additional file 1: **Table S1.** Predesigned TaqMan® Gene Expression assays ^*#*^ Reference genes used to normalize the results. (DOCX 14 kb)

## Abbreviations

ABCG5: ATP binding cassette, sub-family G member 5
ABCG8: ATP binding cassette, sub-family G member 8
ACAT2: Acetyl-Coenzyme A acetyltransferase 2
ApoE^−/−^: Apolipoprotein E-deficient
CE: Cholesteryl esters
CI: Confidence interval
CLO: Cod liver oil
CO: Corn oil
CVD: Cardiovascular disease
CWO: Cold pressed whale oil
CYP7A1: Cytochrome P450 7A1
DHA: Docosahexaenoic acid
DPA: Docosapentaenoic acids
EPA: Eicosapentaenoic acid
FA: Fatty acids
FASN: Fatty acid synthase
HDL: High density lipoproteins
HMGCR: 3-hydroxy-3-methyl-glutaryl-Coenzyme A reductase
HPRT1: Hypoxanthine-guanine phosphoribosyltransferase 1
ICAM1: Intercellular adhesion molecule 1
IFNγ: Interferon gamma
IL-10: Interleukin 10
IL-1β: Interleukin 1 beta
IL-2: Interleukin 2
IL-5: Interleukin 5
IL-6: Interleukin 6
KC-GRO: Keratinocyte chemoattractant growth-regulated oncogene
LC n3-PUFA: Long chain n3-polyunsaturated fatty acid
LDL: Low density lipoprotein
LDLR: LDL-receptor
MCP-1: Monocyte chemotactic protein 1
n3-PUFA: n3-polyunsaturated fatty acid
NEFA: Non-esterified fatty acids
NFE212: Nuclear factor erythroid 2-related factor
Ox-LDL: Oxidized low density lipoprotein
PON2: Paroxynase 2
PPARα: Peroxisome proliferator-activated receptor*-α*
PPARγ: Peroxisome proliferator-activated receptor*-*γ
PUFA: Polyunsaturated fatty acid
RWO: Refined whale oil
RWO-I: Refined whale oil + extract I
RWO-II: Refined whale oil + extract II
SR-B1: Scavenger receptor class B member 1
TAG: Triacylglycerol
TAS: Serum total antioxidant status
TBP: TATA-Box Binding Protein
TNFα: Tumour necrosis factor alpha
UCP2: Uncoupling protein 2
VCAM1: Vascular adhesion molecule 1
VLDL: Very low density lipoprotein
VLDLR: Very low density lipoprotein receptor
WD: Western type diet
