# Supplementary material for: Cold-pressed minke whale oil reduces circulating LDL/VLDL-cholesterol, lipid oxidation and atherogenesis in apolipoprotein E-deficient mice fed a Western-type diet for 13 weeks
Source: Nutr Metab (Lond). 2018 May 4;15:35. doi: 10.1186/s12986-018-0269-8 (PMC5935995; doi:10.1186/s12986-018-0269-8)
Supplement: Supplementary file 1 — Table S1. Predesigned TaqMan® Gene Expression assays # Reference genes used to normalize the results. (DOCX 14 kb) [file 12986_2018_269_MOESM1_ESM.docx]

| **Abbreviation** | **Gene name** | **Assay #** |
| --- | --- | --- |
| *Abcg5* | ATP binding cassette, sub-family G member 5 | Mm00446241_m1 |
| *Abcg8* | ATP binding cassette, sub-family G member 8 | Mm00445980_m1 |
| *Acat2* | Acetyl-Coenzyme A acetyltransferase 2 | Mm00782408_s1 |
| *Cyp7a1* | Cytochrome P450 7A1 | Mm00484150_m1 |
| *Fasn* | Fatty acid synthase | Mm00662319_m1 |
| *Hmgcr* | 3-hydroxy-3-methyl-glutaryl-Coenzyme A reductase | Mm01282499_m1 |
| *Hprt1^#^* | Hypoxanthine-guanine phosphoribosyltransferase | Mm01545399_m1 |
| *Icam1* | Intercellular adhesion molecule 1 | Mm00516023_m1 |
| *Il-6* | Interleukin-6 | Mm00446190_m1 |
| *Ldlr* | LDL-receptor | Mm01177349_m1 |
| *Mcp1* | Monocyte chemotactic protein 1 | Mm00441242_m1 |
| *Nfe212* | Nuclear factor erythroid 2-related factor | Mm00477784_m1 |
| *Pon2* | Paroxynase 2 | Mm00447159_m1 |
| *Pparα* | Peroxisome proliferator-activated receptor*-α* | Mm00440939_m1 |
| *Ppar*γ | Peroxisome proliferator-activated receptor*-*γ | Mm01184322_m1 |
| *Sr-b1* | Scavenger receptor class B member 1 | Mm00450234_m1 |
| *Tbp^#^* | TATA-Box Binding Protein | Mm00446971_m1 |
| *Tnfα* | Tumor necrosis factor-*α* | Mm00443258_m1 |
| *Ucp2* | Uncoupling protein 2 | Mm00627599_m1 |
| *Vcam1* | Vascular adhesion molecule 1 | Mm01320970_m1 |
| *Vldlr* | Very low density lipoprotein receptor | Mm00443298_m1 |
